# Exploring Metabolic Shifts in Kidney Cancer and Non-Cancer Cells Under Pro- and Anti-Apoptotic Treatments Using NMR Metabolomics

**DOI:** 10.3390/cells14050367

**Published:** 2025-03-02

**Authors:** Lucia Trisolini, Biagia Musio, Beatriz Teixeira, Maria Noemi Sgobba, Anna Lucia Francavilla, Mariateresa Volpicella, Lorenzo Guerra, Anna De Grassi, Vito Gallo, Iola F. Duarte, Ciro Leonardo Pierri

**Affiliations:** 1 Department of Biosciences, Biotechnologies and Environment, University of Bari “Aldo Moro”, Via Orabona, 4, 70125 Bari, Italy; lucia.trisolini@uniba.it (L.T.); maria.sgobba@uniba.it (M.N.S.); anna.francavilla@uniba.it (A.L.F.); mariateresa.volpicella@uniba.it (M.V.); lorenzo.guerra1@uniba.it (L.G.); anna.degrassi@uniba.it (A.D.G.); 2 Department of Civil, Environmental, Land, Building Engineering and Chemistry (DICATECh), Polytechnic University of Bari, Via Orabona, 4, 70125 Bari, Italy; biagia.musio@poliba.it (B.M.); vito.gallo@poliba.it (V.G.); 3 CICECO-Aveiro Institute of Materials and LAQV-REQUIMTE, Department of Chemistry, University of Aveiro, 3810-193 Aveiro, Portugal; b.teixeira@ua.pt; 4 Innovative Solutions S.r.l.—Spin-Off Company of the Polytechnic University of Bari, Zona H 150/B, 70015 Noci, Italy; 5 Department of Pharmacy—Pharmaceutical Sciences, University of Bari “Aldo Moro”, Via Orabona, 4, 70125 Bari, Italy

**Keywords:** NMR metabolomics, mitochondria, apoptosis, Staurosporine, Bongkrekic acid, kidney cell lines

## Abstract

This study investigates the metabolic responses of cancerous (RCC) and non-cancerous (HK2) kidney cells to treatment with Staurosporine (STAU), which has a pro-apoptotic effect, and Bongkrekic acid (BKA), which has an anti-apoptotic effect, individually and in combination, using ^1^H NMR metabolomics to identify metabolite markers linked to mitochondrial apoptotic pathways. BKA had minimal metabolic effects in RCC cells, suggesting its role in preserving mitochondrial function without significantly altering metabolic pathways. In contrast, STAU induced substantial metabolic reprogramming in RCC cells, disrupting energy production, redox balance, and biosynthesis, thereby triggering apoptotic pathways. The combined treatment of BKA and STAU primarily mirrored the effects of STAU alone, with BKA showing little capacity to counteract the pro-apoptotic effects. In non-cancerous HK2 cells, the metabolic alterations were far less pronounced, highlighting key differences in the metabolic responses of cancerous and non-cancerous cells. RCC cells displayed greater metabolic flexibility, while HK2 cells maintained a more regulated metabolic state. These findings emphasize the potential for targeting cancer-specific metabolic vulnerabilities while sparing non-cancerous cells, underscoring the value of metabolomics in understanding apoptotic and anti-apoptotic mechanisms. Future studies should validate these results in vivo and explore their potential for personalized treatment strategies.

## 1. Introduction

Kidney cancer, particularly renal cell carcinoma (RCC), is a complex malignancy with significant therapeutic challenges and variable patient outcomes. Effective biomarkers and targeted therapies are crucial for improving diagnosis, treatment, and prognosis in kidney cancer [1,2]. Metabolic profiling is a powerful tool for uncovering biochemical changes associated with cancer and therapeutic responses, providing insights into disease mechanisms and potential treatment targets [3,4].

Apoptosis, or programmed cell death, plays a pivotal role in cancer biology, influencing tumor progression and response to treatment. Staurosporine (STAU) is a potent pro-apoptotic agent that can trigger apoptosis through multiple pathways. At higher concentrations, STAU induces apoptosis by inhibiting various protein kinases, including protein kinase C (PKC), and cyclin-dependent kinases, which disrupt critical signaling pathways and promote cell death. At lower concentrations, STAU can also trigger mitochondrial apoptosis by causing the release of cytochrome c and other pro-apoptotic factors from mitochondria [5,6,7]. This mitochondrial pathway involves the disruption of mitochondrial membrane potential and the activation of apoptosome formation [8,9].

In contrast, Bongkrekic acid (BKA), which has an anti-apoptotic effect, counteracts mitochondrial apoptosis by targeting the ADP/ATP carrier (AAC, also known as ANT) [10,11]. AAC is a key component of the mitochondrial permeability transition pore (mPTP), which regulates the release of pro-apoptotic factors from mitochondria [11,12,13]. By binding to AAC in m-conformation, BKA inhibits mPTP opening, thereby preventing mitochondrial membrane permeabilization and the subsequent release of apoptosis-inducing factors [11,12,13,14,15].

To investigate the metabolic impact of these treatments, we used a a non-cancerous proximal tubular kidney cell line, HK2 [12,16,17], and a kidney cancer cell line, RCC-Shaw, which is derived from primary RCC tissue [12,18,19,20]. The non-cancerous proximal tubular kidney cell line, HK2, provided a baseline for comparative analysis with the RCC cancer cell-line [12,16,17,18,19,20]. These cell lines were treated with STAU, BKA, or a combination of both, with a specific focus on the pre-incubation with BKA followed by STAU treatment to evaluate if BKA can mitigate the apoptotic effects of STAU and to highlight metabolites whose concentration varied in the three abovementioned conditions.

In the last decades, ^1^H NMR metabolomics has been employed to identify biomarkers of all-cause mortality and to monitor prognostic factors in health and disease [18,19]. In the present study, we employed untargeted ^1^H NMR metabolomics for analyzing the intracellular and extracellular metabolites (endo- and exo-metabolome, respectively) affected by the different treatments (BKA, STAU, and BKA+STAU). Our primary aims were to gain insights into the metabolic pathways underlying apoptosis and cell survival, and to identify potential biomarkers associated with metabolic alterations in mitochondrial apoptotic pathways.

## 2. Materials and Methods

### 2.1. Cell Culture

The non-tumor human proximal tubule kidney cells (HK2) were obtained from the American Type Culture Collection (ATCC, Manassas, VA, USA). Renal carcinoma cells (RCC-Shaw) are primary RCC cell lines established from primary kidney tissue explants derived from biopsy and currently provided by Public Health England (PHE)—culture collection (PHE, Salisbury, UK) [20]. HK2 cells were maintained in high-glucose Dulbecco’s Modified Eagle’s Medium (DMEM) with sodium pyruvate and stable glutamine (Euroclone, ECL0103L, Pero, Milan, Italy), supplemented with 10% Fetal Bovine Serum (FBS; Euroclone ECS0180L, Pero, Milan, Italy) and 1% penicillin–streptomycin (Euroclone, ECM0010, Pero, Milan, Italy) [12]. RCC cells were grown in Roswell Park Memorial Institute (RPMI 1640) medium with stable glutamine (Euroclone, ECM2001L, Pero, Milan, Italy) supplemented with 10% FBS (Euroclone ECS0180L, Pero, Milan, Italy) and 1% penicillin–streptomycin (Euroclone, ECM0010, Pero, Milan, Italy) [12]. All cells were cultured in a humidified atmosphere with 5% CO_2_ at 37 °C.

### 2.2. Cell Exposure for Metabolomics Assays

The investigated cell lines, grown on 10 cm-diameter Petri dishes, were treated with a highly selective ADP/ATP carrier (AAC) inhibitor, namely, the anti-apoptotic Bongkrekic acid, indicated as BKA, at 40 μM for 7 h [10,11], or the intrinsic apoptosis initiator Staurosporine, indicated as STAU, at 10 μM for 3 h [21]. In addition, a 4-h-long pre-incubation with BKA (40 μM), followed by the addition of STAU (10 μM for 3 h), condition was tested. A complete culture medium without molecules was added to control cells. Three independent assays with two replicates each were performed, giving a total of six samples per condition.

### 2.3. Extraction of Intracellular and Extracellular Metabolites

For metabolomics, water-soluble intracellular metabolites were extracted using a dual-phase extraction procedure adapted from Teng et al. [22]. The culture medium was collected, and each dish was washed twice using PBS (Euroclone, ECB4004, Pero, Milan, Italy). To halt metabolic activity, 650 μL of cold methanol 80% was added to the cells, which were then scraped off the dish and vortexed for 1 min in microcentrifuge tubes. To facilitate cell lysis, the samples were subjected to three freeze–thaw cycles, alternating between liquid nitrogen and 37 °C. Next, 260 μL of chloroform (added twice) and 220 μL of water were sequentially added to each sample, with 1 min of vortexing after each addition. The samples were incubated on ice for 10 min and then centrifuged at 2000× *g* for 15 min. The upper aqueous phase was carefully transferred into new vials, dried under vacuum, and stored at −80 °C until analysis. For medium samples, proteins were depleted by centrifuging at 1000× *g* for 5 min, adding cold methanol (1:2, *v*/*v*), incubating for 30 min at −20 °C, and centrifuging at 13,000× *g* for 20 min. The resulting supernatant was dried under vacuum. Before analysis, the dried polar extracts and medium samples were reconstituted in 600 μL of deuterated phosphate buffer (100 mM, pH 7.4) containing 0.1 mM of TSP-*d*_4_, and 550 μL of the reconstituted solution was transferred into 5 mm NMR tubes for analysis.

### 2.4. NMR Data Acquisition and Processing

^1^H NMR spectra were acquired on a Bruker Avance III HD spectrometer operating at 500.13 MHz for ^1^H observation, at 298 K, using a 5 mm TXI probe. Standard 1D ^1^H spectra with water pre-saturation (pulse program “noesypr1d” from Bruker library) were recorded with a 7002.8 Hz spectral width, 32 k data points, a 4 s relaxation delay, and 512 scans. Spectral processing was carried out in TopSpin 4.0.3 (Bruker BioSpin, Rheinstetten, Germany), using cosine multiplication (ssb 2), zero-filling to 64 k data points, manual phasing, baseline correction, and calibration to the TSP-*d*_4_ signal at 0 ppm. Metabolites were identified by matching the obtained spectral data to reference spectra in the reference libraries in the Human Metabolome Database (HMDB), BBIOREFCODE-2–0–0 (Bruker Biospin, Rheinstetten, Germany), and Chenomx NMR Suit version 8.5 (Edmonton, AB, Canada) [23].

### 2.5. Multivariate and Univariate Statistical Analysis

Using the Amix-Viewer software (version 3.9.15, Bruker Biospin, Rheinstetten, Germany), the spectra were segmented into regular-sized (0.005 ppm) intervals (buckets) and normalized by total signal area (excluding suppressed water and residual solvent signals). The resulting data matrices were uploaded into SIMCA-P 11.5 (Umetrics, Sweden) and scaled to unit variance. Principal component analysis (PCA) and partial least squares-discriminant analysis (PLS-DA) were then applied. The results were visualized through factorial coordinates (scores) and contributions (loadings), which were color-coded based on variable importance to the projection (VIP). Loadings profiles were generated using the R software version 4.1.3 (R Core Team (2020). R: A language and environment for statistical computing. R Foundation for Statistical Computing, Vienna, Austria. URL: http://www.R-project.org/, accessed on 18 December 2024).

To provide a quantitative measurement of metabolic variations, selected signals were integrated and normalized by total area using the Amix-Viewer software (version 3.9.15). The magnitude of metabolite changes was assessed through the percentage of variation (and its respective error) in treated samples compared to controls. Statistical significance was assessed using the Student’s *t*-test for comparisons between two groups and one-way ANOVA followed by Dunnett’s test for multiple comparisons, performed using the Prism software (version 8.0.2, GraphPad, La Jolla, CA, USA). A *p*-value < 0.05 was considered statistically significant.

## 3. Results

### 3.1. Comparison of RCC and HK2 Metabolic Profiles

**Figure 1:**
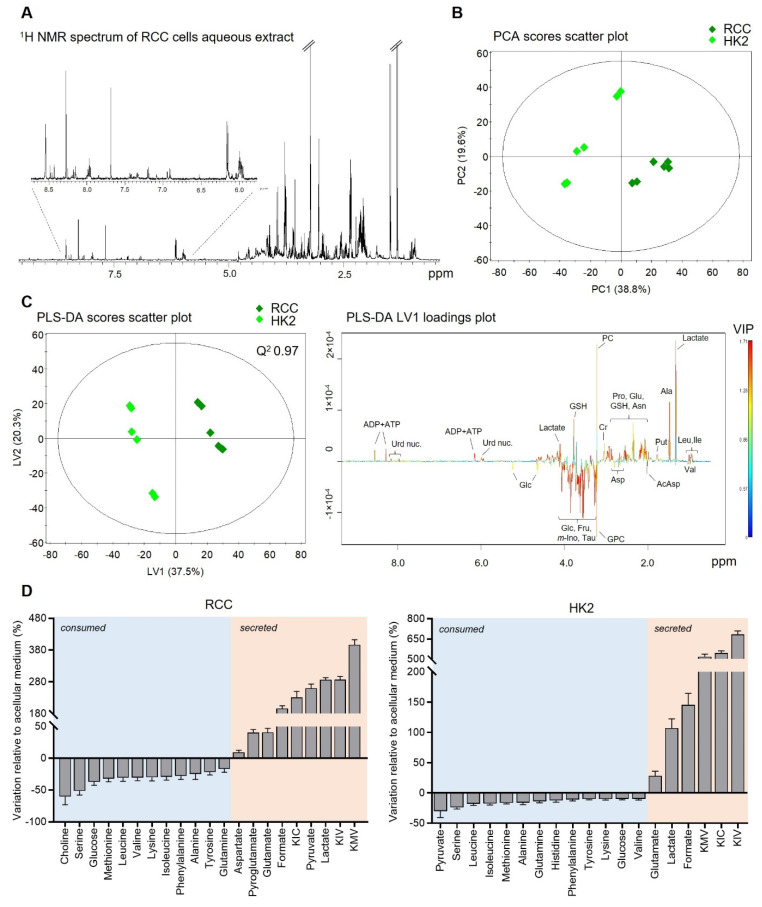
NMR metabolic profile of RCC and HK2 cells: (**A**) Representative ^1^H NMR spectrum of RCC cells’ aqueous extracts. (**B**) Scores scatter plot obtained by PCA of ^1^H NMR spectra from RCC and HK2 cells’ aqueous extracts. (**C**) Scores scatter plot (**left**) and LV1 loadings (**right**) obtained by PLS-DA of ^1^H NMR spectra from RCC and HK2 cells’ aqueous extracts. (**D**) Metabolite consumption and secretion patterns of RCC (**left**) and HK2 cells (**right**) expressed as % variation relative to acellular medium. Abbreviations: AcAsp, N-acetylaspartate; ADP/ATP, adenosine di/triphosphate; Cr, creatine; Fru, fructose; Glc, glucose; GPC, glycerophosphocholine; GSH, glutathione; m-Ino, myo-inositol; PC, phosphocholine; Put, putrescine; Urd nuc., uridine nucleotides; Tau, taurine. Three-letter codes were used for amino acids.

The ^1^H NMR spectrum of the cell’s aqueous extracts (Figure 1A) revealed signals corresponding to over 30 intracellular metabolites. A complete list of identified metabolites and their ^1^H NMR resonances can be found in Supplementary Table S1. In the low-frequency region (δ 0.5–3.0 ppm), signals from various amino acids (e.g., leucine, isoleucine, valine, alanine, threonine, glutamate, glutamine, aspartate, and asparagine), glutathione (GSH), and organic acids, such as acetate and lactate, were identified. The mid-frequency region (δ 3.0–5.5 ppm) contained signals from metabolites, including creatine, phosphocreatine, choline, phosphocholine, taurine, myo-inositol, and sugars (glucose and fructose). In the high-frequency region (δ 5.5–9.0 ppm), signals related to aromatic amino acids (tyrosine, phenylalanine, and histidine) and nucleotides (e.g., ADP, ATP, and NAD^+^) were observed.

The spectral profiles of RCC and HK2 cells’ aqueous extracts were distinctly separated by PCA, as shown in Figure 1B. PLS-DA (Figure 1C) further validated the robust discrimination between the two cell types (Q^2^ 0.97), with the LV1 loadings highlighting the primary metabolic differences. Quantitative analysis (Supplementary Table S2) confirmed significant metabolic differences between RCC and non-cancerous HK2 cells. RCC cells exhibited markedly higher levels of 16 metabolites, including lactate, citrate, formate, creatine, phosphocreatine, uridine nucleotides, ADP+ATP, GSH, phosphocholine, putrescine, and some amino acids (asparagine, alanine, leucine, isoleucine, proline, and glutamate). Conversely, HK2 cells showed higher levels of glucose, fructose, myo-inositol, taurine, glycerophosphocholine (GPC), pantothenate, and other amino acids, such as aspartate, N-acetylaspartate, threonine, valine, glycine, phenylalanine, and β-alanine.

The consumption and secretion patterns of the two cell lines also displayed notable differences (Figure 1D and Supplementary Table S3). RCC cells consumed over 50% of the extracellular levels of choline and serine, 30–50% of glucose, branched-chain amino acids (BCAAs), methionine, and lysine, and 15–30% of glutamine, alanine, and aromatic amino acids. In contrast, HK2 cells showed the highest consumption of pyruvate (−30.1 ± 10.7%), followed by several amino acids and glucose (−9.9 ± 1.4%). Both cell types secreted glutamate, formate, branched-chain α-ketoacids (BCKAs), and lactate, albeit to differing extents. Notably, lactate was the primary metabolite secreted by RCC cells, whereas HK2 cells released higher amounts of BCKAs. Additionally, pyruvate, which was consumed by HK2 cells, was secreted by RCC cells.

### 3.2. Impact of BKA, STAU, and BKA+STAU Treatments on RCC Cells

**Figure 2:**
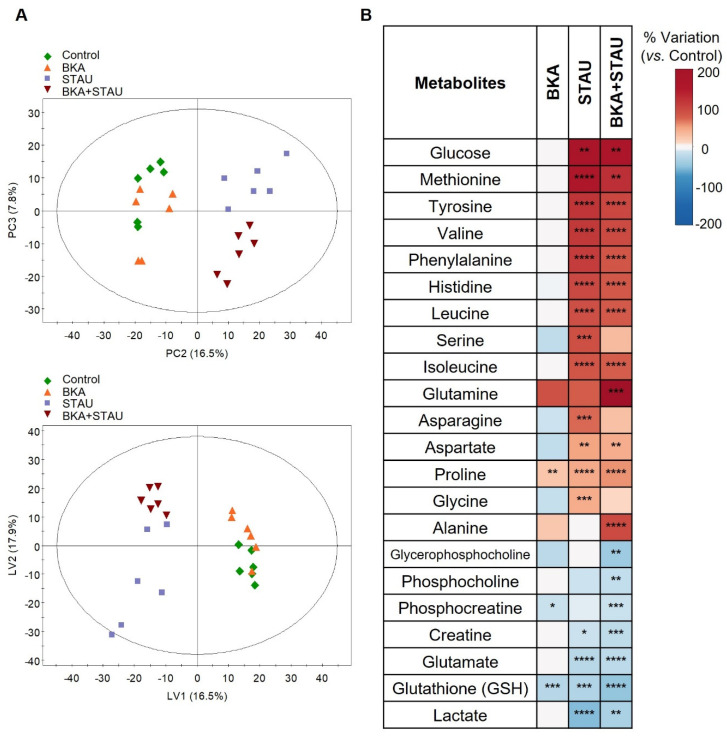
Impact of BKA, STAU, and BKA+STAU treatments on RCC cells’ intracellular metabolites: (**A**) Scores scatter plots obtained by applying PCA (**top**) and PLS-DA (**bottom**) to ^1^H NMR spectra of RCC cells’ aqueous extracts. (**B**) Heatmap summarizing the intracellular polar metabolite levels in RCC-treated cells, expressed as % of variation relative to untreated controls. Statistical significance was assessed with respect to untreated controls (* *p* < 0.05, ** *p* < 0.01, *** *p* < 0.005, and **** *p* < 0.001).

To investigate the impact of different treatments on RCC cells’ intracellular metabolites, a two-step analysis was performed, beginning with multivariate analysis (MVA) applied to the ^1^H NMR spectra of cells’ aqueous extracts. The PC2 vs. PC3 scores scatter plot (Figure 2A, top) provided the clearest separation between the sample groups. Untreated controls and BKA-treated cells clustered together in negative PC2, whereas cells treated with STAU or BKA+STAU were positioned in positive PC2, with further separation along PC3. Notably, STAU-treated and BKA+STAU-treated cells were distinct from each other. This group differentiation was largely preserved in the PLS-DA scores plot (Figure 2A, bottom), which also demonstrated moderate discrimination between untreated controls and BKA-treated cells.

Building on these observations, spectral signal integration was conducted to quantify relative variations in intracellular polar metabolites of treated RCC cells relative to controls. The quantitative results, presented in Figure 2B and Supplementary Table S4, provide further insights into the metabolic shifts induced by the treatments. Consistent with the MVA findings, BKA treatment induced relatively few changes in the metabolic composition of RCC cells. The only significant differences observed compared to controls were elevated levels of proline and reduced levels of phosphocreatine and GSH. In contrast, treatment with STAU markedly altered the levels of 17 intracellular metabolites. Notably, glucose and 11 amino acids exhibited significantly elevated levels in STAU-treated RCC cells compared to untreated controls. Conversely, the intracellular levels of lactate, glutamate, creatine, phosphocreatine, choline-containing compounds, and GSH were significantly decreased following STAU treatment. Interestingly, most of these metabolic changes were preserved in cells pre-incubated with BKA prior to STAU treatment (BKA+STAU group), suggesting that BKA pre-treatment was ineffective in preventing or counteracting the effects of STAU. However, exceptions were observed for serine, asparagine, and glycine, whose increases were not detected in cells treated with both drugs. Additionally, BKA+STAU-treated cells exhibited further reductions in phosphocreatine and choline-containing compounds compared to controls, along with more pronounced increases in glutamine and alanine levels.

**Figure 3:**
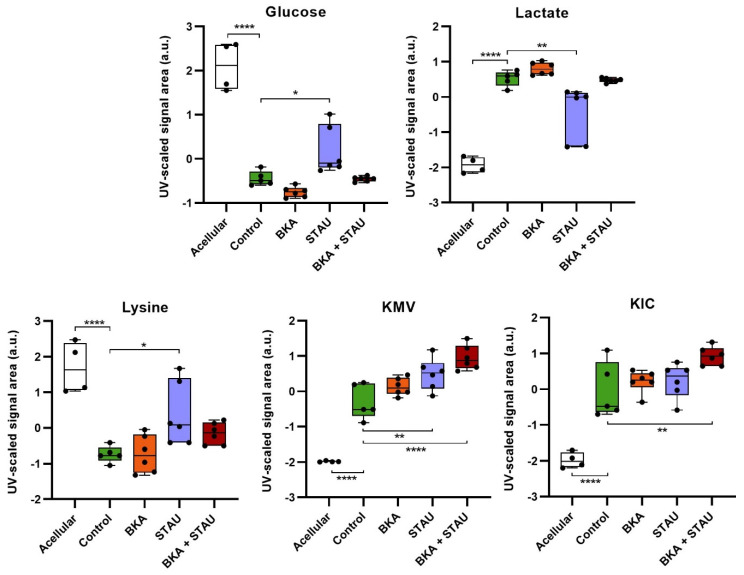
Impact of BKA, STAU, and BKA+STAU treatments on RCC cells’ extracellular metabolites: Relative metabolite levels in acellular medium and in medium conditioned by untreated (Control) and treated cells, as assessed by spectral integration followed by total area normalization and scaling to unit variance. Statistical significance was assessed with respect to untreated controls (* *p* < 0.05, ** *p* < 0.01, and **** *p* < 0.001).

At the exometabolome level (Figure 3), the impact of the treatments was less pronounced, likely due to the short incubation period of 7 h. Nevertheless, some changes were observed: STAU treatment resulted in reduced glucose and lysine consumption, accompanied by decreased lactate secretion. Additionally, cells treated with STAU and/or BKA+STAU exhibited increased secretion of BCKAs.

### 3.3. Impact of BKA, STAU, and BKA+STAU Treatments on HK2 Cells

**Figure 4:**
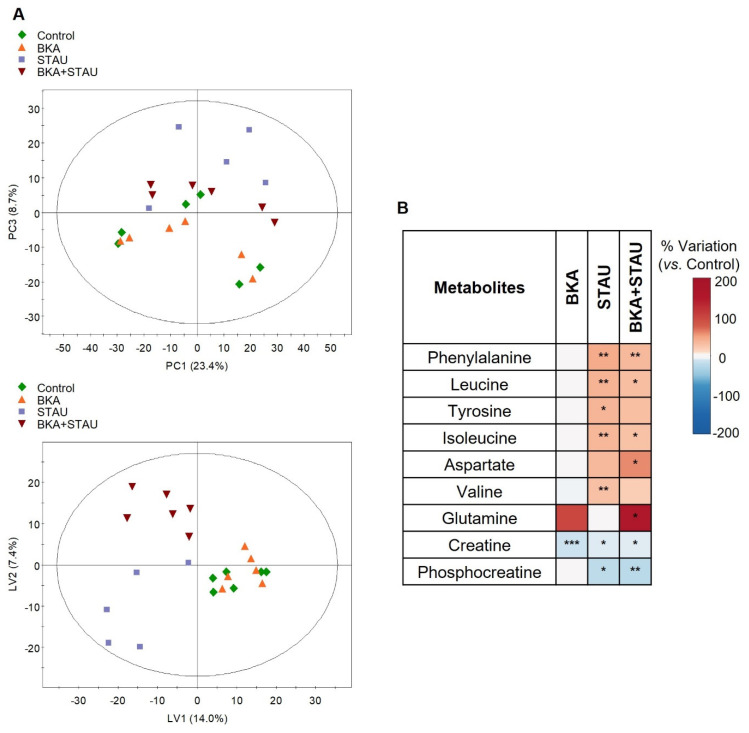
Impact of BKA, STAU, and BKA+STAU treatments on HK2 cells’ intracellular metabolites: (**A**) Scores scatter plots obtained by applying PCA (**top**) and PLS-DA (**bottom**) to ^1^H NMR spectra of HK2 cells’ aqueous extracts. (**B**) Heatmap summarizing the variations in treated cells vs. controls (the metabolites included showed at least one statistically significant variation relative to controls, as assessed by ANOVA, * *p* < 0.05, ** *p* < 0.01, and *** *p* < 0.005).

PCA of the ^1^H NMR spectra from non-cancerous HK2 cells did not demonstrate a clear separation between the control and treated sample groups. However, a trend was observed, with STAU- and BKA+STAU-treated cells displaying some differentiation from the other two groups along PC3 (Figure 4A, top). Sample group discrimination was further improved by PLS-DA, although untreated controls and BKA-treated cells remained closely clustered, indicating their similar metabolic profiles (Figure 4A, bottom).

Quantitative analysis of intracellular metabolites confirmed that the treatments had a low (BKA) to mild (STAU and BKA+STAU) impact on HK2 cells (Figure 4B and Supplementary Table S5). BKA treatment resulted in a significant decrease in creatine levels. STAU and BKA+STAU treatments further reduced phosphocreatine levels compared to controls and altered the levels of certain amino acids, including BCAAs and aromatic amino acids. The combined treatment (BKA+STAU) also significantly increased the levels of aspartate and glutamine. Regarding extracellular metabolites, none of the treatments resulted in significant differences compared to the controls.

## 4. Discussion

This study provides insights into the metabolic responses of kidney cells to pro-apoptotic (STAU) and anti-apoptotic (BKA) treatments, individually and in combination, emphasizing the identification of biomarkers linked to mitochondrial apoptotic pathways. By employing ^1^H NMR metabolomics, distinct metabolic adaptations were observed in RCC and HK2 cell lines, reflecting their differential susceptibilities and metabolic reprogramming under apoptotic and anti-apoptotic conditions.

**Figure 5:**
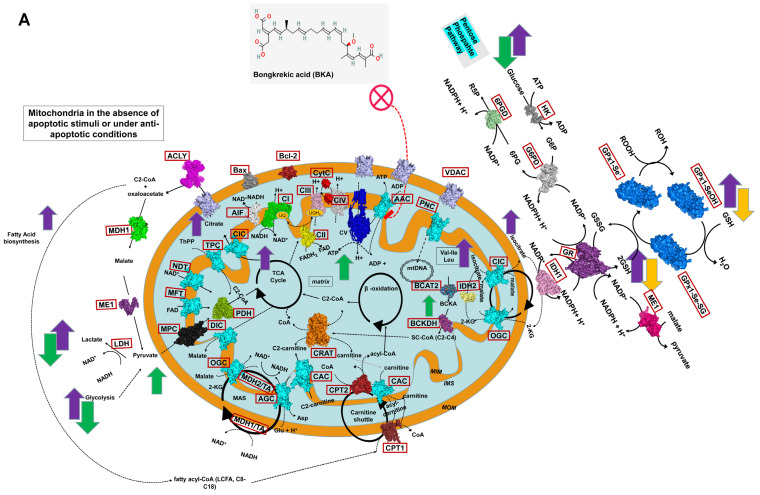

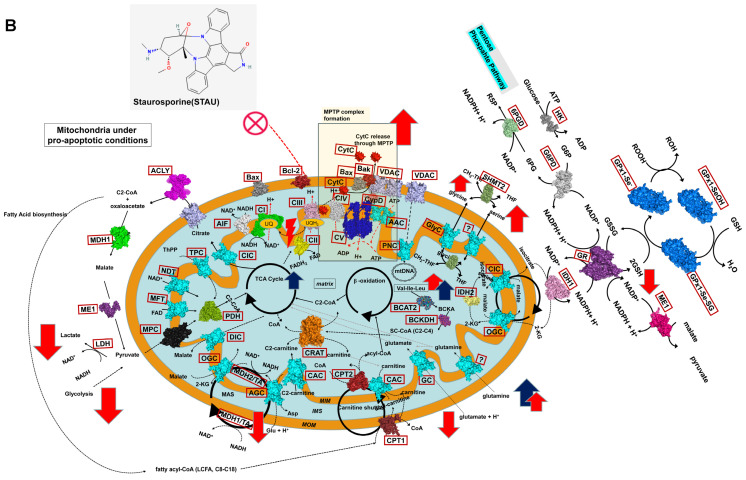
Schematic representation of mitochondria under anti-apoptotic and pro-apoptotic stimuli. (**A**) Mitochondria in cells under basal conditions (without apoptotic stimuli) or treated with the anti-apoptotic molecule BKA. The schematic highlights representative proteins, pathways, and cycles. Green arrows (up or down) indicate increased or decreased consumption or production of specific metabolites in untreated HK2 cells, respectively. The orange down-arrow indicates decreased metabolite consumption or production in RCC cells treated with BKA, while the violet up-arrow denotes increased metabolite consumption or production in untreated RCC cells. (**B**) Mitochondria in cells exposed to pro-apoptotic stimuli, specifically STAU or BKA+STAU, which induce the formation of the mitochondrial permeability transition pore (mPTP). Red arrows (up or down) indicate increased or decreased consumption or production of metabolites in RCC cells treated with STAU, respectively, while the dark-blue up-arrow indicates increased consumption or production of metabolites in RCC cells treated with BKA+STAU. Proteins are shown in surface representations using PyMOL, with atomic coordinates derived from the Protein Data Bank (PDB). Protein names are reported in brown boxes for discriminating them from substrates. ATP synthase (CV) is represented in blue (6zqn.pdb), CRAT in orange (1nm8.pdb), BCAT2 in blue–green (5mpr.pdb), and BCKDH in dark magenta–blue (1u5b.pdb). CPT1 and CPT2 are shown in dark violet (4ep9.pdb), and VDAC is represented in pink (2jk4.pdb). Bax and Bak/Bcl-2 are depicted in dark grey and firebrick, respectively (4s0o.pdb, 2yv6.pdb). MPC is shown in black based on an in-house 3D comparative model, PDH is in light green (6cfo.pdb), and AIF is in white (4bur.pdb). Respiratory complexes I–IV are visualized as follows: CI in green (5lnk.pdb), CII in yellow (3aef.pdb), CIII in magenta (6q9e.pdb), and CIV (together with CytC in red) in grey (5iy5.pdb). The 3D comparative models of mitochondrial carriers of the SLC25A family are shown in cyan (based on the bovine ADP/ATP carrier structure 1okc.pdb). Black circular arrows represent cyclic pathways, while black solid and dashed lines indicate reaction directions. Magenta dashed lines highlight the administration of anti-apoptotic (BKA) or pro-apoptotic (STAU) small molecules. Abbreviations: C2-CoA, acetyl-CoA; C2-carnitine, acetyl-carnitine; SC-CoA, short-chain acyl-CoA; LCFA, long-chain fatty acids; BCFA, branched-chain fatty acids; BCKA, branched-chain ketoacids; MIM, mitochondrial inner membrane; MOM, mitochondrial outer membrane; IMS, intermembrane space; UQ, ubiquinone; AAC, ADP/ATP carrier (SLC25A4/5/6/31); TPC, thiamine pyrophosphate carrier (SLC25A19); CAC, carnitine/acyl-carnitine carrier (SLC25A20); AGC, aspartate/glutamate carrier (SLC25A12 and SLC25A13); DIC, dicarboxylate carrier (SLC25A10); NDT, NAD+ carrier (SLC25A51); MFT, FAD carrier (SLC25A32); OGC, malate/2-oxoglutarate carrier (SLC25A11); CIC, citrate carrier (SLC25A1); PiC, phosphate carrier (SLC25A3); CoAC, CoA carrier (SLC25A42); MAS, malate-aspartate shuttle; TCA, tricarboxylic acid cycle; Bax, Bcl-2-associated X protein; Bak, Bcl-2 antagonist/killer-1; Bcl-2, B-cell lymphoma-2; MDH1, cytosolic malate dehydrogenase 1; ME1, malic enzyme 1; MPC, mitochondrial pyruvate carrier; PDH, pyruvate dehydrogenase; CypD, cyclophilin D; CytC, cytochrome C; VDAC, voltage-dependent anion channel; AIF, apoptosis-inducing factor; PNC, pyrimidine nucleotide carrier (SLC25A33 and SLC25A36).

The first key finding of this study was the distinct metabolic phenotypes of RCC and HK2 cells, which reflected adaptations to their different functional and physiological roles, as well as the altered metabolic requirements of cancer cells compared to non-cancerous cells (Figure 5A). RCC cells exhibited elevated lactate levels and increased lactate secretion, consistent with the Warburg effect, a hallmark of cancer metabolism. This metabolic reprogramming shifts cellular energy production toward glycolysis, even in the presence of oxygen, resulting in lactate accumulation [12,24,25,26]. In addition, RCC cells showed higher consumption of glucose, branched-chain amino acids (BCAAs), and other amino acids, indicating their reliance on glycolysis and amino acid metabolism to fuel energy production and anabolic pathways for rapid growth and proliferation (Figure 5A). In contrast, HK2 cells primarily utilized pyruvate and secreted elevated amounts of branched-chain α-ketoacids (BCKAs), reflecting active BCAA catabolism for energy generation (Figure 5A). RCC cells, however, retained more BCAAs, likely redirecting them toward biosynthesis and tricarboxylic acid (TCA) cycle replenishment. Higher levels of citrate, formate, and proline in RCC cells suggest enhanced TCA cycle activity and one-carbon metabolism. Citrate contributes to lipid biosynthesis, while formate supports nucleotide synthesis and epigenetic regulation. Proline metabolism further fuels TCA cycle intermediates, promoting mitochondrial activity and biosynthesis. The elevated levels of GSH, ADP+ATP, creatine, and phosphocreatine in RCC cells indicate increased energy demand and a need for redox homeostasis. GSH plays a crucial role in antioxidant defense, protecting cancer cells from oxidative stress [27,28,29], while the creatine–phosphocreatine system buffers ATP levels during rapid energy fluctuations, enabling sustained high metabolic activity [30,31]. Additionally, the significant consumption of choline and increased phosphocholine levels in RCC cells underscore their dependence on choline metabolism for membrane biosynthesis and signaling, processes that are often upregulated in cancer cells to support rapid proliferation [32,33].

Overall, RCC cells demonstrated a broader substrate utilization profile, consuming serine, choline, and aromatic amino acids alongside glucose and BCAAs. This metabolic flexibility enables RCC cells to adapt to varying nutrient availability and maintain anabolic processes. In contrast, HK2 cells exhibited a more balanced metabolic phenotype, characterized by lower glucose consumption, substantial pyruvate utilization, and reduced lactate secretion. These traits indicate a reliance on oxidative phosphorylation (OXPHOS) rather than glycolysis. Furthermore, higher levels of glucose, fructose, and other amino acids in HK2 cells suggest a more conservative metabolic approach, reflecting their homeostatic, non-cancerous state.

It should also be mentioned that RCC cells and non-cancerous HK2 cells exhibit distinct redox balances, with RCC cells often displaying elevated basal oxidative stress due to metabolic reprogramming and altered mitochondrial function [34,35]. In contrast, HK2 cells possess a more robust antioxidant defense, including higher glutathione levels and superoxide dismutase activity, which helps maintain redox homeostasis and protect against oxidative damage [36,37]. Oxidative stress plays a crucial role in mitochondrial apoptosis regulation by promoting mitochondrial outer membrane permeabilization and mPTP opening [38]. While our study focused on the direct effects of STAU and BKA on mPTP regulation, future investigations should assess the antioxidant status of these cell lines under our experimental conditions to better understand its potential role in modulating mPTP dynamics and apoptotic susceptibility.

The response of the two cell lines to the anti- and pro-apoptotic treatments was significantly different, with the impact being much more pronounced in RCC cells (Figure 5B). It should be noticed that BKA is a selective, non-competitive inhibitor of the ADP/ATP carrier, able to block the carrier from the matrix face, preventing the formation of the mitochondrial permeability transition pore [10,11,13,39,40,41,42,43]. Conversely, STAU, a broad-spectrum kinase inhibitor, triggers both intrinsic (mitochondrial) and extrinsic (receptor-mediated) apoptotic pathways, with a concentration-dependent effect. At lower concentrations (e.g., 10 µM), it predominantly activates mitochondrial apoptosis by modulating the expression of Bcl-2 family proteins [44], leading to cytochrome c release and caspase-9 activation [7,12,21,43,45]. It should be noticed that STAU can also modulate Bcl-2 family proteins, leading to mitochondrial outer membrane permeabilization (MOMP) through mechanisms beyond the full assembly of the mPTP [46]. Additionally, it has been proposed that the mPTP can function in at least two main modes: a high-conductance state, associated with prolonged pore opening and sustained ∆Ψm dissipation, and a low-conductance state, characterized by transient pore openings that result in mitochondrial depolarization spikes [13,47,48]. While multiple proteins, including members of the Bcl-2 family, have been implicated in regulating mitochondrial permeabilization, the ADP/ATP carrier (AAC, also known as ANT) has been identified as a key regulator of the low-conductance state opening [10,12,13,49,50,51]. In light of these observations, while BKA effectively targets the ADP/ATP carrier and prevents the low-conductance state formation and related mPTP-dependent apoptosis, it may not fully counteract STAU-induced cell death if additional mitochondrial destabilization pathways, such as MOMP via Bax/Bak activation, are engaged [46]. At higher concentrations, STAU can also stimulate extrinsic apoptosis via activation of death receptor pathways, further amplifying caspase cascades. This dual capability underscores its potent pro-apoptotic properties across various cell types [52].

Concerning BKA, which is able to prevent the formation of the low-conductance-state formation and the ADP/ATP carrier-mediated mitochondrial permeability transition pore formation [7,12,21,43,45], its administration to RCC cells induced relatively minor changes in the intracellular metabolome. The elevated proline and glutamine levels may reflect a stress-related adaptation, as both amino acids are often associated with oxidative stress responses and redox regulation [53,54] (Figure 5A). The decrease in phosphocreatine and GSH levels suggests a mild disruption in energy buffering and antioxidant defense mechanisms, which are critical for maintaining cellular homeostasis. However, the minimal metabolic shifts indicate that BKA primarily succeeds in preserving mitochondrial integrity without significantly altering the overall metabolic state of RCC cells under the studied conditions (Figure 5A).

Conversely, STAU caused substantial metabolic reprogramming in RCC cells, consistent with its role in inducing apoptosis [12,21,55,56]. The observed increase in glucose and amino acid levels (serine, glycine, and asparagine) could indicate an apoptotic response involving the accumulation of biosynthetic precursors due to impaired utilization (Figure 5B). Reduced levels of lactate point to a suppression of glycolysis, possibly reflecting a metabolic shift away from the Warburg effect (Figure 5B). This is corroborated by lower glucose consumption and lactate secretion. Similarly, the depletion of glutamate, creatine, phosphocreatine, and GSH underscores a disruption in energy production, redox balance, and osmotic regulation, hallmarks of apoptosis. The decline in choline-containing compounds likely reflects altered membrane metabolism and a breakdown of phospholipid components, consistent with apoptotic membrane remodeling.

The combined treatment of BKA and STAU largely mirrored the metabolic profile of STAU-treated RCC, suggesting that BKA was ineffective in counteracting the pro-apoptotic effects of STAU. Notable exceptions included the absence of increases in serine, asparagine, and glycine, which might point to a partial modulation of amino acid metabolism by BKA at the employed concentration. However, further reductions in phosphocreatine- and choline-containing compounds, along with intensified increases in glutamine and alanine, highlighted enhanced metabolic disruption under the combined treatment (Figure 5B). Enhanced secretion of BCKAs was also observed in BKA+STAU-treated cells, suggesting enhanced catabolism of branched-chain amino acids, possibly to fuel the TCA cycle or generate substrates for redox balance and biosynthesis during apoptosis (Figure 5B). These changes suggest that pre-incubation with BKA may exacerbate certain apoptotic metabolic alterations in RCC.

The alterations observed in non-cancerous HK2 cells in response to the treatments were much less pronounced than those in RCC cells, highlighting key differences in the metabolic adaptability of cancerous versus non-cancerous cells. The muted response of HK2 cells reflects their stable, tightly regulated metabolic network, in contrast to the metabolic flexibility of RCC cells, which rely on altered pathways to sustain rapid proliferation and evade apoptosis. For instance, RCC cells’ heavy reliance on glycolysis, evidenced by elevated lactate production and glucose consumption, makes them more susceptible to treatments targeting these pathways. In contrast, HK2 cells maintain a more balanced metabolic state that does not depend on glycolysis or other cancer-specific mechanisms, allowing them to resist metabolic disruptions. The differential response to treatments also underscores the importance of mitochondrial function and metabolic flexibility in cancer cell survival. The significant changes induced by STAU in RCC cells align with their heightened mitochondrial activity, including oxidative phosphorylation and the tricarboxylic acid cycle. Conversely, the limited effect of BKA on HK2 cells suggests that, unlike RCC cells, these cells are less dependent on anti-apoptotic mechanisms tied to mitochondrial metabolism.

## 5. Conclusions

This study demonstrated that BKA and STAU, both individually and in combination, induced distinct metabolic shifts associated with the modulation of mitochondrial apoptotic pathways. STAU profoundly impacted RCC cell metabolism, disrupting energy production, redox balance, and biosynthesis, which in turn drove apoptotic pathways. In contrast, BKA had a limited metabolic effect at the employed concentration and did not counteract the changes induced by STAU. The differential metabolic responses of HK2 and RCC cells emphasized the contrasting metabolic landscapes of non-cancerous and cancerous cells. RCC cells underwent significant metabolic reprogramming in response to the treatments, reflecting their dependence on altered metabolic pathways for survival and proliferation. In contrast, HK2 cells exhibited metabolic stability, with only modest changes in response to the same treatments. This underscores the potential of targeting cancer-specific metabolic vulnerabilities at the level of mitochondrial pathways, while minimizing effects on non-cancerous cells.

This work represents the first report on the effects of anti- and pro-apoptotic agents on kidney cells, highlighting the potential of metabolomics to reveal the biochemical adaptations underlying apoptosis-related mechanisms. These insights lay a foundation for advancing therapeutic strategies aimed at targeting mitochondrial pathways. Future research should focus on validating these findings in vivo and exploring their translational potential for personalized treatment strategies.

## Data Availability

The data that support the findings of this study are available from the corresponding author upon reasonable request. Data will be made available upon request.

## Supplementary Materials

The following supporting information can be downloaded at: https://www.mdpi.com/article/10.3390/cells14050367/s1. Table S1: Metabolites identified in the aqueous extracts of HK2 and RCC cells using ^1^H NMR; Table S2: Percentual variation (% ± error) of the levels of intracellular polar metabolites in untreated RCC cells relatively to non-cancerous untreated HK2 cells. Variations between −5 and 5% and those for which the error was larger than the variation module were considered null; Table S3. Variations in extracellular metabolites of untreated RCC and HK2 cells relative to respective acellular media (% ± error); Table S4: Percentual variation (% ± error) of the levels of intracellular polar metabolites in treated RCC cells relatively to untreated controls. Variations between −5 and 5% and those for which the error was larger than the variation module were considered null; Table S5. Percentual variation (% ± error) of the levels of intracellular polar metabolites in treated HK2 cells relatively to untreated controls. Variations between −5 and 5% and those for which the error was larger than the variation module were considered null.
