# Supplementary material for: Exploring Metabolic Shifts in Kidney Cancer and Non-Cancer Cells Under Pro- and Anti-Apoptotic Treatments Using NMR Metabolomics
Source: Cells. 2025 Mar 2;14(5):367. doi: 10.3390/cells14050367 (PMC11899725; doi:10.3390/cells14050367)
Supplement: Supplementary file 1 [file cells-14-00367-s001.zip › cells-3467306-supplementary.pdf]

Supplementary Information

Exploring Metabolic Shifts in Kidney Cancer and Non-Cancer Cells Under Pro- and Anti-Apoptotic Treatments Using NMR Metabolomics

Authors

Lucia Trisolini [1,†], Biagia Musio [2,†], Beatriz Teixeira [3], Maria Noemi Sgobba [1], Anna Lucia Francavilla [1], Mariateresa Volpicella [1], Lorenzo Guerra [1], Anna De Grassi [1], Vito Gallo [2,4], Iola F. Duarte [3,*] and Ciro Leonardo Pierri [5,*]

Affiliations

[1] Department of Biosciences, Biotechnologies and Environment, University of Bari "Aldo Moro", Via Orabona, 4, 70125 Bari, Italy
[2] Department of Civil, Environmental, Land, Building Engineering and Chemistry (DICATECh), Polytechnic University of Bari, Via Orabona, 4, 70125 Bari, Italy
[3] CICECO-Aveiro Institute of Materials and LAQV-REQUIMTE, Department of Chemistry, University of Aveiro, 3810-193 Aveiro, Portugal
[4] Innovative Solutions S.r.l.—Spin-Off Company of the Polytechnic University of Bari, Zona H 150/B, 70015 Noci, Italy
[5] Department of Pharmacy—Pharmaceutical Sciences, University of Bari "Aldo Moro", Via Orabona, 4, 70125 Bari, Italy

* Correspondence: ioladuarte@ua.pt (I.F.D.); ciro.pierri@uniba.it (C.L.P.)
[†] These authors contributed equally to this work.

Table S1. Metabolites identified in the aqueous extracts of HK2 and RCC cells using ^{1}H NMR.

No.	Metabolites	δ^1-H in ppm (multiplicity, assignment)
1	Acetate	1,92 (s, β-CH_3)
2	ADP+ATP	4,24 (m, C5'H, ribose); 4,28 (m, C5''H, ribose); 4,40 (m, C4'H, ribose); 4,57 (m, C2'H, ribose); 6,15 (d, C1'H, ribose); 8,25 (s, C2, ring), 8,54 (s, NH, ring)
3	Alanine	1.49 (d, β-CH_3); 3.79 (m, α-CH)
4	Asparagine	2.86 (dd, β-CH); 2.96 (dd, β'-CH); 4.00 (dd, α-CH)
5	Aspartate	2.68 (dd, β-CH); 2.82 (dd, β'-CH); 3.89 (dd, α-CH)
6	Choline	3.21 (s, $N(CH_3)_3$); 3.53 (m, β-CH); 4.07 (d, α-CH_2)
7	Creatine	3.04 (s, CH_3); 3.93 (s, CH_2)
8	Formate	8.46 (s, CH)
9	Fumarate	6.52 (s, CH)
10	α-Glucose	3,42 (m, C4H); 3.54 (dd, C2H); 3.70 (m, C3H); 3.82 (m, C6H); 3.85 (m, C5H); 5.24 (d, C1H)
11	β-Glucose	3.25 (dd, C2H); 3.41 (m, C4H); 3.47 (m, C5H); 3.48 (t, C3H); 3.76 (m, C6H); 3.90 (dd, C6'H); 4.65 (d, C1H)
12	Glutamate	2.06 (m, β-CH); 2.14 (m, β'-CH); 2.35 (m, γ-CH_2); 3.76 (t, α-CH)
13	Glutamine	2.13 (m, β-CH_2); 2.46 (m, γ-CH_2); 3.78 (t, α-CH)
14	Glutathione	2.17(m, β-CH_2, Glu); 2.56 (m, γ-CH_2, Glu); 2.96 (m, β-CH_2, Cys); 3.77 (m, α-CH); 4.57 (m, α-CH, Cys)
15	Glycerophosphocholine	3.23 (s, $N(CH_3)_3$); 3.68 (m, β'-CH); 4.33 (m, α'-CH_2)
16	Glycine	3.56 (s, α-CH_2)
17	Histidine	3,13 (dd, β-CH_2); 3.24 (dd, β'-CH_2); 3.98 (dd, α-CH); 7.09 (s, C4H, ring); 7.87 (s, C2H, ring)
18	Isoleucine	0.94 (t, δ-CH_3); 1.02 (d, β'-CH_3); 1.26 (m, γ-CH_2); 1.48 (m, γ'-CH_2); 1.99 (m, β-CH_2); 3.68 (d, α-CH)
19	Lactate	1.33 (d, β'-CH_3); 4.11 (m, α-CH)
20	Leucine	0.96 (d, δ-CH_3); 0.97 (d, δ'-CH_3); 1.68 (d, β'-CH_3); 1.71 (m, γ-CH); 1.75 (m, β-CH_2); 3.74 (t, α-CH)
21	Methionine	2.13 (s, $CH_3(S)$); 2.20 (m, β-CH_2); 2.65 (t, γ-CH); 3.87 (m, α-CH)
22	myo-Inositol	3.28 (t, C5H); 3,54 (C1H, C3H); 3.63 (dd, C4H); 4.07 (t, C2H)
23	NAD^+	4,27 (m, A5'); 4.37 (m, A4'); 4.38 (m, A4'/N5'); 4.42 (dd, N3'); 4.51 (m, A3'); 4.55 (m, N2'); 6.04 (d, N1'); 6.09 (d, A1'); 8.18 (s, A2); 8.20 (t, N5), 8.43 (s, A8); 8.84 (d, N4); 9.15 (d, N6); 9.34 (s, N2)
24	Phenylalanine	3.13 (m, β-CH); 3.29 (dd, β'-CH); 4.00 (m, α-CH); 7.33 (d, C2H, C6H, ring); 7.38 (d, C4H, ring); 7.43 (t, C3H, ring)
25	Phosphocholine	3.23 (s, $N(CH_3)_3$); 3.60 (m, N-CH_2); 4.17 (m, PO_3-CH_2)
26	Phosphocreatine	3.04 (s, CH_3); 3.95 (s, CH_2)
27	Proline	2.00 (m, γ-CH_2); 2.05 (m, β-CH); 2.34 (m, β'-CH); 3.34 (dt, δ-CH); 3.42 (dt, δ'-CH); 4.14 (dd, α-CH)
28	Putrescine	1.78 (m, $(CH_2)_2$); 3.05 (m, N-CH_2)
29	Serine	3.85 (dd, α-CH); 3.97 (m, β-CH_2)
30	Taurine	3.27 (t, S-CH_2); 3.43 (t, N-CH_2)
31	Threonine	1.33 (d, γ-CH_3); 3.59 (d, α-CH); 4.25 (m, β-CH)
32	Tyrosine	3.06 (m, β'-CH); 3.20 (m, β-CH); 3.94 (m, α-CH); 6.91 (d, C3H, C5H, ring); 7.20 (d, C2H, C6H, ring)
33	Uridine nucleotides	3.89 (dd, C5'H, ribose); 4.13 (m, C4'H, ribose); 4.24 (t, C3'H, ribose); 4.36 (t, C2'H, ribose); 5.98 (d, C5H, ring); 6.00 (d, C1'H, ribose); 7.96 (d, C6H, ring);
34	Valine	1.00 (d, γ-CH_3); 1.05 (d, γ'-CH_3); 2.28 (m, β-CH); 3.60 (m, α-CH)

Table S2. Percentual variation (% ± error) of the levels of intracellular polar metabolites in untreated RCC cells relatively to non-cancerous untreated HK2 cells. Variations between -5 and 5% and those for which the error was larger than the variation module were considered null.

	**%**	**error**	**p-value**
Fructose	-91.73	8.40	3.71E-06
Glucose	-90.16	53.84	2.81E-02
myo-Inositol	-80.42	8.98	1.08E-05
Taurine	-70.80	11.88	2.84E-05
Aspartate	-68.36	29.83	1.61E-02
Threonine	-64.88	16.23	1.58E-03
Pantothenate	-60.34	8.62	5.01E-06
N-Acetylaspartate	-53.14	4.18	9.29E-06
Glycine	-48.94	15.05	6.08E-03
β-Alanine	-46.49	21.85	2.48E-02
Glycerophosphocholine	-45.67	14.74	3.31E-03
Valine	-31.08	5.45	3.89E-04
Phenylalanine	-28.59	9.51	1.17E-02
Creatine	8.87	3.55	3.91E-02
Phosphocreatine	19.34	5.00	6.89E-03
Phosphocholine	23.89	4.51	8.72E-04
Urdnucleotides	27.53	6.05	2.71E-03
Glutamate	30.31	6.77	9.71E-03
Glutathione	32.31	6.04	1.60E-03
Proline	48.37	7.28	1.23E-03
Leucine	51.15	4.64	9.31E-06
Alanine	65.34	9.48	4.27E-04
Isoleucine	67.41	4.91	1.33E-06
ADP+ATP	84.41	5.98	1.64E-05
Formate	95.16	12.10	1.25E-03
Putrescine	114.35	9.33	8.20E-05
Citrate	130.56	6.01	8.97E-06
Lactate	166.39	10.88	1.30E-04
Asparagine	4989.47	15.50	5.73E-05

Table S3. Variations in extracellular metabolites of untreated RCC and HK2 cells relative to respective acellular media (% ± error).

	RCC			HK2		
	**%**	**error**	**p-value**	**%**	**error**	**p-value**
Choline	-60.37	12.68	5.03E-04	n.s.	-	-
Serine	-51.54	6.46	1.63E-05	-23.89	2.79	4.96E-04
Glucose	-37.19	5.24	1.69E-03	-9.87	1.39	1.10E-03
Methionine	-31.92	5.04	7.90E-04	-16.44	1.67	3.60E-04
Leucine	-30.75	5.50	3.22E-03	-17.89	2.51	2.87E-04
Valine	-30.18	5.22	1.61E-03	-9.86	1.89	1.58E-03
Lysine	-30.08	5.53	4.54E-03	-10.09	1.74	2.41E-03
Isoleucine	-29.15	5.03	3.04E-03	-17.57	2.34	1.95E-04
Phenylalanine	-27.73	5.69	1.34E-03	-10.87	2.17	4.09E-03
Alanine	-24.75	8.59	1.24E-02	-16.31	2.93	1.33E-03
Tyrosine	-21.71	4.58	8.22E-03	-10.11	1.26	1.01E-03
Glutamine	-16.92	5.16	2.64E-02	-13.81	2.44	4.27E-03
Aspartate	9.36	3.29	4.14E-02	n.s.	-	-
Pyroglutamate	40.61	4.52	2.11E-04	n.s.	-	-
Glutamate	40.81	6.24	1.39E-03	28.49	7.66	3.35E-02
Formate	196.46	8.39	2.08E-04	145.76	18.79	1.80E-02
KIC	231.15	19.01	3.64E-03	545.26	15.27	1.48E-03
Pyruvate	260.09	12.87	2.75E-04	-30.11	10.70	2.43E-02
Lactate	286.39	6.72	1.56E-06	107.23	15.16	8.68E-03
KIV	287.35	9.35	2.87E-05	683.77	25.19	5.48E-03
KMV	396.52	16.26	1.57E-03	518.22	18.80	2.17E-03
Histidine	n.s.	-	-	-12.53	2.65	2.83E-03

n.s. not significant

Table S4. Percentual variation (% ± error) of the levels of intracellular polar metabolites in treated RCC cells relatively to untreated controls. Variations between -5 and 5% and those for which the error was larger than the variation module were considered null.

	**BKA**		**STAU**		**BKA+STAU**	
	**%**	**error**	**%**	**error**	**%**	**error**
Glucose	0	-	215.20	29.57	189.47	21.14
Methionine	0	-	178.99	19.98	124.37	7.41
Tyrosine	0	-	118.38	11.31	98.86	4.89
Valine	0	-	114.52	14.02	95.71	3.95
Phenylalanine	0	-	111.82	12.27	84.79	4.40
Histidine	-5.88	5.87	97.29	12.02	80.54	5.41
Leucine	0	-	91.60	13.18	79.89	4.89
Serine	-19.91	5.39	90.75	18.90	32.60	4.60
Isoleucine	0	-	83.56	11.72	77.27	4.68
Glutamine	89.41	36.05	77.90	56.26	328.89	14.08
Asparagine	-12.14	11.63	71.57	13.97	29.51	8.27
Aspartate	-19.53	11.62	49.61	11.27	43.36	10.74
Proline	25.55	6.84	45.29	5.60	56.14	4.15
Glycine	-17.27	8.80	43.74	8.48	11.98	5.30
Alanine	23.98	7.79	0	-	95.54	4.17
Glycerophosphocholine	-23.65	16.47	0	-	-42.15	15.28
Phosphocholine	0	-	-12.57	6.58	-18.65	4.67
Phosphocreatine	-16.90	5.94	-12.96	5.77	-44.14	5.67
Creatine	0	-	-12.99	2.72	-20.97	4.78
Glutamate	0	-	-25.64	5.72	-22.66	2.18
Glutathione	-25.93	8.10	-30.05	5.18	-48.31	4.81
Lactate	0	10.48	-54.08	11.21	-34.55	9.26

Table S5. Percentual variation (% ± error) of the levels of intracellular polar metabolites in treated HK2 cells relatively to untreated controls. Variations between -5 and 5% and those for which the error was larger than the variation module were considered null.

	**BKA**		**STAU**		**BKA+STAU**	
	**%**	**error**	**%**	**error**	**%**	**error**
Phenylalanine	0	-	44.05	11.94	34.75	8.25
Leucine	0	-	38.96	10.72	30.90	8.34
Tyrosine	0	-	37.57	13.33	30.22	9.68
Isoleucine	0	-	35.89	10.76	27.81	7.33
Aspartate	0	-	34.46	20.71	59.30	15.34
Valine	0	-	28.63	9.06	19.98	7.09
Glutamine	100.63	40.51	0	-	163.41	33.37
Creatine	-12.42	3.21	-8.13	3.49	-7.86	3.18
Phosphocreatine	0	-	-20.93	5.75	-23.96	5.98
